# Rumen Fermentation and Microbiome Responses to Enzymatic Hydrolysate of Cottonseed Protein Supplementation in Continuous In Vitro Culture

**DOI:** 10.3390/ani12162113

**Published:** 2022-08-18

**Authors:** Jia Zhou, Ziyue Ding, Qijian Pu, Benchu Xue, Shuangming Yue, Shengtao Guan, Zhisheng Wang, Lizhi Wang, Quanhui Peng, Bai Xue

**Affiliations:** 1 Animal Nutrition Institute, Sichuan Agricultural University, Chengdu 611130, China; 2 Chengdu Mytech Biotech Co., Ltd., Chengdu 611130, China; 3 Department of Bioengineering, Sichuan Water Conservancy College, Chengdu 611845, China

**Keywords:** enzymatic hydrolysate of cottonseed protein, in vitro fermentation, gas production, rumen fermentation characteristics, microbial diversity

## Abstract

**Simple Summary:**

The enzymatic hydrolysate of cottonseed protein (ECP) is a product rich in small chain peptides obtained from cottonseed meal by enzymatic hydrolysis. The removal of gossypol and the high content of protein and small chain peptides make ECP a potentially superior additive to cow feed. In this study, we employed the technique of in vitro gas production to simulate rumen fermentation and found that ECP supplementation increased the cumulative gas production, concentrations of ammonia nitrogen, and microbial proteins but did not affect the composition and production of volatile fatty acids. Compared with the positive control (supplemented yeast culture), the effect of ECP supplementation on the structure and composition of bacteria is much smaller. The findings of this study suggest that ECP is a high-quality additive that can be added to the feed of cows.

**Abstract:**

This study aimed to evaluate the effect of enzymatic hydrolysate of cottonseed protein (ECP) on the kinetic of gas production, rumen fermentation characteristics, and microbial diversity in continuous in vitro culture with a single factorial design of supplementation with various concentrations of ECP or yeast culture. Treatments were control (without supplementation, CON), supplementation with 10 g/kg Diamond-V XP yeast culture of substrate (XP), and supplementation with 6, 12 and 18 g/kg ECP of substrate (ECP1, ECP2, ECP3), each incubated with 30 mL of buffered incubation fluids and 200 mg of fermentation substrate in graduated glass syringes fitted with plungers for 48 h. Compared with the CON treatment, supplementation of XP yeast culture increased the cumulative gas production at 12 and 24 h, the concentration of ammonia nitrogen (NH_3_-N) concentration at 24 and 36 h, the concentration of microbial protein (MCP) concentration at 24 and 48 h, the molar butyrate proportion at 12, 24, and 48 h, the molar valerate proportion at 48 h, and the ratio of non-glucogenic to glucogenic acids (*p* < 0.05). Compared with the CON treatment, the concentration of MCP and the molar propionate proportion at 12 h were higher in the ECP1 treatment (*p* < 0.05); the cumulative gas production at 2, 4, and 12 h, the concentration of NH_3_-N at 36 h and the molar valerate proportion at 48 h were higher in the ECP2 treatment (*p* < 0.05); the cumulative gas production at 2, 12, and 48 h, the concentration of NH_3_-N at 12 and 36 h, the concentration of MCP at 12, 36, and 48 h, the molar butyrate proportion at 12 and 48 h, and the molar valerate proportion at 48 h were higher in the ECP3 treatment (*p* < 0.05). Compared with the CON treatment, supplementation with XP yeast culture significantly altered the relative abundance of the phyla Firmicutes, Kiritimatiellaeota, and Proteobacteria, while supplementation with ECP had minimal effect on bacterial diversity. The prediction of bacterial functions showed that the main gene functions of rumen bacteria are associated with carbohydrate metabolism, amino acid metabolism, and membrane transport. The findings of this study suggest that ECP can be used as a superior feed ingredient for ruminants, the suitable level of ECP was 18 g/kg in vitro experiment.

## 1. Introduction

Besides production efficiency, the physiological functions and health of livestock are attracting more and more attention [1]. Nutritional strategies have emerged and it has been proposed as a key factor to improve the health status and welfare of animals as well as to enhance productivity [2,3]. Modulation of fermentation parameters by supplementing additives to ruminant diets was considered an effective strategy for manipulating rumen function [4,5].

The shortage of feed ingredients and the high price of imported protein sources are the main factors restricting the further development of animal husbandry in China [6]. Especially in the last few years, the price of soybean meal, the major source of protein for livestock feed, has increased substantially, as well as the cost of feedstuff [7]. Cottonseed meal, the main by-product of the cottonseed oil extraction, contains more than 40% of crude protein (CP) and has been considered as a cheap substitute for soybean meal. However, due to the existence of toxic free gossypol, the proportion of cottonseed meal in animal feed is limited [8]. For adult ruminants, although the mature microbial system in the rumen is able to convert free gossypol into protein-gossypol complexes, it is not absorbed in the digestive tract thereby hindering its entry into the blood [9]. Excessive free gossypol intake could escape this protective mechanism and increase gossypol content in the plasma and milk of cows [10], induce a negative nitrogen balance in lactating goats [11], and disrupt the integrity of sperm cell membrane in male ruminants [12]. Moreover, the high content of free gossypol had obvious negative effects on both microbial diversity and activity [13,14]. In addition to containing toxic free gossypol, the high fiber, low lysine and methionine contents, and lower digestibility of cottonseed meal limit its use in growing livestock [15,16]. Based on these, cottonseed meal has not been fully utilized at present.

Several methods have been proven to reduce free gossypol content in cottonseed meal. Solvent extraction could reduce free gossypol content to less than 0.045%, making cottonseed meal an edible high-quality protein [17]. The free gossypol in cottonseed meal could be degraded to less than 0.005% by microbial (*C. tropicalis* or *S. cerevisae*) fermentation [18]. Enzymolysis is a method of applying protease to reduce free gossypol, while hydrolyzing macromolecular proteins to peptides to improve the nutritional quality of cottonseed meal [19,20]. Earlier studies revealed that rumen microbes utilize both ammonia nitrogen (NH_3_-N) and non-ammonia nitrogen (e.g., free amino acids and peptides) to synthesize microbial protein (MCP), and some of them preferentially use non-ammonia nitrogen over NH_3_-N [21,22]. Supplementation of peptides enhanced the efficiency of rumen microbe growth, ruminal fermentation, and fiber digestion in vitro [23,24], and nitrogen utilization efficiency in vivo [25]. Except for utilization by rumen microbes, small peptides obtained from proteolysis of plant proteins can be directly absorbed by the rumen epithelium, thereby more conducive to retaining their original biological activities including improving immunological and antioxidant status [26,27]. Enzymolysis would greatly improve the availability of cottonseed meal to alleviate the current shortage of protein resources. The addition of 2% enzymatic hydrolysate of cottonseed protein (ECP) in a starter diet enhanced the antioxidant status of newborn calves, whereas 6% ECP supplementation impaired the starter intake and growth of newborn calves and exposed them to a stressful status [15]. However, the evaluation of the nutritional value of ECP as a feed material for adult ruminants has not yet been reported.

Yeast culture is a unique yeast product due to the composition of yeasts and their metabolites, and it maintains the fermentation activity of yeast [28]. The composition and characteristics of these metabolites are determined by multiple factors during yeast cultivation, e.g., culture medium, yeast species, and culture conditions [29]. Functional metabolites in yeast cultures may contribute to rumen function by providing beneficial nutrients otherwise scarce in the ruminal environment [30]. Previous studies have shown that yeast culture had a positive effect on rumen fermentation and microbial metabolism [31,32]. We hypothesized that supplementation with different levels of ECP had no negative effect on rumen fermentation. In this study, yeast culture was used as a positive control and the primary objective was to investigate the in vitro dynamic changes of ruminal fermentation characteristics, kinetic of gas production, and microbial diversity with different levels of ECP supplementation.

## 2. Materials and Methods

### 2.1. Materials

The ECP used in this experiment was a commercial product, Fortide^®^, prepared from cottonseed meal and by enzymatic hydrolysis into small chain peptides (Chengdu Mytech Biotech Co., Ltd., Chengdu, China). The nutritional values (dry matter (DM)) of ECP were as follow: CP > 48.9%, crude fiber < 7.6%, dietary peptides (molecular weights: 1.0–5.0 kDa) > 30.4%, according to the manufacturer’s instructions. Diamond-V XP yeast culture (Diamond V Mills, Inc., Cedar Rapids, IA, USA) consists of *Saccharomyces cerevisiae*, corn protein feed, wheat cereals, rye middlings, and molasses, and the minimum content of CP was 13.5% (DM).

### 2.2. Experimental Design

The rumen fermentation characteristics with the addition of different levels of ECP were determined in a continuous culture in vitro with a single factorial design of supplementation with various concentrations of ECP or yeast culture. Based on the difference in additions, there were five treatments as follow: control (without supplementation, CON), Diamond-V XP yeast culture supplementation (10 g/kg of fermentation substrate, as a positive control group, XP), and ECP supplementation (6, 12, and 18 g/kg of fermentation substrate, ECP1, ECP2, and ECP3, respectively). Ingredients and chemical composition of the fermentation substrate are shown in Table 1. Gas production and fluid samples with three replicates for each treatment were counted and collected at 2, 4, 8, 12, 24, 36, and 48 h. A part of fluid samples (6 mL) collected at 24 h were used to microbial community analyses. All experimental protocols were approved by the Animal Ethical and Welfare Committee (AEWC) of Animal Nutrition Institute, Sichuan Agricultural University (No. SCAU202010-3).

### 2.3. In Vitro Fermentation

According to the method described by Menke [33], in vitro fermentation was performed in graduated glass syringes fitted with plungers (volume capacity of 100 mL, Changzhou Wuxing Medical Instrument Co., Ltd., Changzhou, China). In brief, rumen fluid was collected from three healthy Holstein cows (dry-period, body weight 624.7 ± 6.8 kg) with a clean catheter connected to a vacuum pump prior to morning feeding. The rumen fluids obtained from three cows, filtered through 4 layers of sterile gauze into a preheated holding bottle, were transported to the laboratory as soon as possible. Equal volumes of three rumen fluids were mixed evenly at 39 °C and purged continuously with CO_2_. The buffer solution was prepared according to Menke and Steingass [34] and maintained at 39 °C by water bath, with removal of air under a continuous flow of CO_2_. Subsequently, 200 mg of fermentation substrate was accurately weighed and added to glass syringes, followed by 30 mL of medium (incubation fluids), consisting of 10 mL of rumen fluids and 20 mL buffer solution [34]. The syringes were incubated in a shaking water bath at 39 °C for 2, 4, 8, 12, 24, 36, and 48 h [35]. To correct for the gas production resulting from the activity of the rumen fluids, three empty syringes only containing equal amounts of incubation medium were incubated as blanks [35].

### 2.4. Gas Production Parameters, Ammonia Nitrogen, Microbial Protein, and Volatile Fatty Acids Measurements

Cumulative gas production was determined by reading the moving scale on the glass syringe plunger after 2, 4, 8, 12, 24, 36, and 48 h of incubation. Two incubation fluid samples (2 mL and 3 mL) in each syringe at 8, 12, 24, 36, and 48 h were collected to determine the concentration of NH_3_-N and MCP, respectively. The incubation fluid samples (2 mL) at 12, 24, and 48 h were collected to determine the content of volatile fatty acids (VFAs). The determination methods of NH_3_-N, MCP, and VFAs in the incubation fluid were performed as previously reported. In brief, the concentration of NH_3_-N concentration was spectrophotometrically measured by the phenol–hypochlorite method with a UV-3600i Plus (Shimadzu, Kyoto, Japan), according to the procedures described by Whitehead [36]. Microbial protein was isolated following the method of Makkar et al. [37] and quantified with a commercial BCA protein assay kit (Nanjing Jiancheng Bio Inst., Nanjing, China), according to the manufacturer’s guidelines. The concentrations of VFAs were analyzed with a V arian CP-3800 gas chromatography (Agilent Technologies Inc., Santa Clara, CA, USA) following the procedures described by Hu et al. [38]. A HP-FFAP capillary column (30 m length × 530 μm inner diameter with 1 μm stationary film thickness, Agilent) was used for chromatographic separation. The oven temperature was held at 100 °C for 2 min, 100 to 190 °C at a rate of 20 °C/min, and held at 190 °C for 5 min. Nitrogen was used as the carrier gas at a flow rate of 1 mL/min.

### 2.5. 16S rRNA Gene Sequencing of Microbes

The total genomic DNA of microbes in incubation fluids at 24 h was extracted using an isolation kit (Tiangen Biotech Co., Ltd., Beijing, China) and then detected by 0.8% agarose gel electrophoresis (Solaibao Technology Co., Ltd., Beijing, China). Targets in the v4 hypervariable regions of bacterial 16S rRNA genes were amplified by 515F (5′-GTGCCAGCMGCCGCGGTAA-3′) and 806R (5′-GGACTACHVGGGTWTCTAAT-3′) [39] with the high-fidelity polymerase KOD-401B FX Neo (Toyobo, Osaka, Japan) in the GeneAmp PCR System 9700 (Applied Biosystems, Foster City, CA, USA). The PCR conditions were as follows: 94 °C for 1 min; followed by 30 cycles of 94 °C for 20 s, 54 °C for 30 s, and 72 °C for 30 s; and a final extension at 72 °C for 5 min. Amplicon sequencing was conducted to generate overlapping paired-end sequencing (2 × 300 bp) on an Illumina HiSeq 2500 platform at Chengdu Rhonin Biosciences Co., Ltd. (Chengdu, China).

### 2.6. Bioinformatics and Analyses

The paired-end sequencing reads obtained from the Illumina platform were merged with the Fast Length Adjustment of SHort reads (FLASH v1.2.7, a freely available as open-source code at https://www.geneious.com/plugins/flash/, accessed on 24 December 2021) and assigned to samples based on their unique barcode [40]. The raw data were generated by intercepting the barcode sequences and trimmed with Trimmomatic v0.36 (http://www.usadellab.org/cms/?page=trimmomatic, accessed on 24 December 2021) [41]. Then, Uchime algorithm (http://drive5.com/usearch/manual/uchime_algo.html, accessed on 24 December 2021) was used to identify and remove chimera sequences based on the “Gold” database (http://drive5.com/uchime/uchime_download.html, accessed on 24 December 2021), resulting in clean reads [42]. The qualified reads were clustered into operational taxonomic units (OTUs) based on the similarity threshold of 97% with Usearch (a free unique sequence analysis tool, http://drive5.com/uparse/, accessed on 25 December 2021) [43]. The analysis of alpha diversity (including observed species, Chao1, Simpson, Shannon index and abundance based coverage estimators (ACE)) and beta diversity, and Venn, rarefaction curves, and Shannon–Wiener curves were carried out with R packages [44,45]. We performed the prediction of functional profiles from 16S rRNA sequencing data with Tax4Fun (a free software package that predicts the functional capabilities of microbial communities based on 16S rRNA datasets, http://tax4fun.gobics.de/, accessed on 22 April 2022) and the SILVA SSU rRNA database [46] and clustered the secondary metabolic pathways.

### 2.7. Calculations and Statistical Analyses

The dynamics parameters of in vitro gas production over time were calculated by following Equation (1), according to Groot et al. [47]:

GP = A/[1 + (C/t)^B^], (1)

where GP was defined as cumulative gas production (mL/0.2 g DM) at time point t (incubation time). The values of “A”, “B”, and “C” were constants of the exponential equation, where “A” is the asymptotic gas production (mL/0.2 g DM), “B” is a sharpness parameter determining the shape of the curve, and “C” is the time (h). The values of “A”, “B”, and “C” were calculated with the non-linear procedure of SPSS v19.0 (SPSS Inc., Chicago, IL, USA). Ulteriorly, we calculated the time corresponding to the maximum rate of gas production (TRmaxG, h), the maximum gas production rate (RmaxG, mL/h) by Equations (2) and (3), according to Yang et al. [48] and the average gas production rate at the time when half of “a” occurred (RahG, mL/h) by Equation (4), according to Chen et al. [49]:

TRmaxG = C × [(B − 1)/(B + 1)]^(1^^/B)^, (2)

RmaxG = (A × C^B^ × b × TRmaxG^(−B − 1)^)/(1 + C^B^ × TRmaxG^(^^−B)^)^2^ (3)

RahG = A × B/(4 × C)(4)

Furthermore, we calculated the ratio of non-glucogenic to glucogenic acids (NGR) by Equation (5), according to Ørskov et al. [50]:

NGR = (acetate + 2 × butyrate + valerate)/(propionate + valerate)(5)

where VFAs were expressed in molar proportion. The appearance of valerate in the numerator and denominator is because valerate may generate one mol of acetate and one mol of propionate upon oxidation [49].

All statistical tests were performed with GLM model for ANOVA analysis by SPSS v19.0 (SPSS Inc., Chicago, IL, USA), followed by Duncan’s multiple range test or Kruskal–Wallis test to compare the differences among the treatment groups. The linear and quadratic effects of ECP supplementation were assessed by curve estimation. Each individual syringe was regarded as a statistical unit, and significance was declared at *p* < 0.05. Results are presented as mean and standard error.

Spearman correlation coefficients (r) and *p* value were analyzed using the OmicShare tools (a free online platform for data analysis, https://www.omicshare.com/tools, accessed on 13 May 2022) to show correlations between gas production and rumen fermentation parameters with bacterial abundances in in vitro fermentation.

## 3. Results

### 3.1. Effect of ECP Supplementation on In Vitro Gas Production and Kinetic Parameters

The cumulative gas production was higher in the EPC2 treatment than in the CON, XP, ECP1, and ECP2 treatments at 2 and 4 h (*p* < 0.05) and increased linearly and quadratically with the ECP supplementation at 2 h (*p* < 0.05). The cumulative gas production was higher in the ECP2, ECP3, and XP treatments than in the CON and ECP1 treatments at 12 h (*p* < 0.05), and increased linearly and quadratically with the ECP supplementation (*p* < 0.05). The cumulative gas production was higher in the XP treatment than in the other four treatments at 12 h (*p* < 0.05) and was not affected by ECP supplementation compared with the CON treatment. The cumulative gas production at 48 h was higher in the ECP3 treatment than in the CON treatment (*p* < 0.05) and increased linearly with the ECP supplementation (*p* < 0.05). The kinetic parameters of gas production were not affected by supplementation, compared with the CON treatment (Table 2).

### 3.2. Effect of ECP Supplementation on Ammonia Nitrogen and Microbial Protein

As shown in Table 3, the concentration of NH_3_-N at 24 h was the highest value in the XP treatment (*p* < 0.05) and was not affected by ECP supplementation compared with the CON treatment. The concentration of NH_3_-N at 12 and 36 h were the highest value in the ECP3 treatment and increased linearly and quadratically with the ECP supplementation (*p* < 0.05). The concentration of MCP at 12 h was higher in the ECP1 and ECP3 treatments than in the CON treatment (*p* < 0.05) and increased linearly and quadratically with the ECP supplementation (*p* < 0.05). The concentration of MCP at 24 h was the highest value in the XP treatment (*p* < 0.05) and was not affected by ECP supplementation compared with the CON treatment. The concentration of MCP at 36 and 48 h were higher in the ECP3 treatment than in the CON treatment (*p* < 0.05) and increased linearly and quadratically with the ECP supplementation (*p* < 0.05).

### 3.3. Effect of ECP Supplementation on Volatile Fatty Acids

The molar acetate proportion at 12 and 48 h were lower in the XP treatment than in the CON treatment (*p* < 0.05) and was not affected by ECP supplementation at 48 h compared with the CON treatment (Table 4). The molar propionate proportion was higher in the ECP1 treatment than in the CON, ECP2, and ECP3 treatments (*p* < 0.05). The molar propionate proportion at 12 and 24 h were the highest value in the ECP1 treatment. The molar butyrate proportion at 12, 24, and 48 h were higher in the XP treatment than in the CON treatment (*p* < 0.05). The molar butyrate proportion at 12 and 48 h were higher in the ECP3 treatment than in the CON treatments (*p* < 0.05) and increased linearly and quadratically with the ECP supplementation (*p* < 0.05). The molar valerate proportion at 48 h were higher in the XP, ECP2, and ECP3 treatments than that in the CON treatment (*p* < 0.05) and increased linearly and quadratically with the ECP supplementation (*p* < 0.05). The concentration of total VFAs at 12 h was lower in the XP treatment than in the other four treatments (*p* < 0.05) and was not affected by ECP supplementation compared with the CON treatment. The ratio of acetate to propionate at 12 h was the lowest value in the XP and ECP1 treatments (*p* < 0.05). Compared with the CON treatment, the value of NGR at 12 h was lower in the ECP1 treatment (*p* < 0.05), and the value of NGR at 24 h was higher in the XP treatment (*p* < 0.05). The value of NGR at 12 and 24 h decreased quadratically with the ECP supplementation (*p* < 0.05).

### 3.4. Operational Taxonomic Unit Diversity of Sequencing

**Figure 1:**
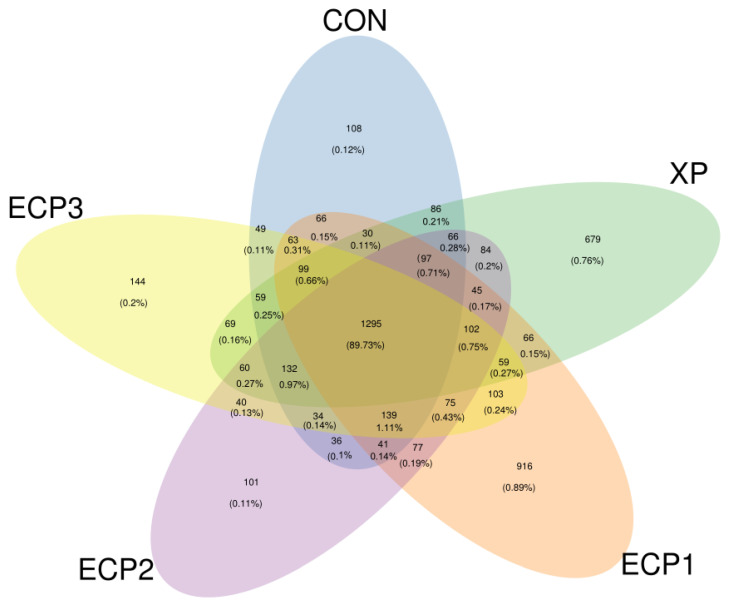
Venn diagram at the 97% sequence similarity of effective sequences. CON treatment, without supplementation; XP treatment, XP yeast culture supplementation with 10 g/kg of substrate; ECP1, ECP supplementation with 6 g/kg of substrate, ECP2, ECP supplementation with 12 g/kg of substrate, ECP3, ECP supplementation with 18 g/kg of substrate.

A total of 553,559 raw sequences in incubation fluids were obtained with 502,917 effective sequences based on the high-throughput sequencing analysis of 16S rRNA genes, the average effective ratio reached 90.08% (Supplementary Table S1). According to the 97% sequence similarity of effective sequences, we obtained 29,575 OTUs in which the average OTUs in the CON, XP, ECP1, ECP2, and ECP3 treatments were 1764, 1806, 2184, 2342, and 1761, respectively (Table 5). There were 1295 OTUs shared across the five treatments, and the number of sequences in shared OTUs accounted for 89.73% of the total number of sequences (Figure 1). The rarefaction curve finally leveled off (Supplementary Figure S1), with a Q30 value (99.9% correct identification of representative bases) greater than 93% (Supplementary Table S1), indicating the ability to detect most bacteria.

### 3.5. Alpha Diversity and Beta Diversity Analyses

Alpha diversity indexes, including the Chao1 and Shannon indexes, were similar among the five treatments (Table 5), implying that there was no difference in the total number of bacteria. The Simpson index in the XP treatment was lower than that in the CON, ECP1, and ECP3 treatments (*p* < 0.05), indicating the lowest richness and most dispersed distribution of bacteria in the XP treatment. Compared with the control group, supplementation of ECP had no significant effect on the diversity of bacterial flora in incubation fluids.

**Figure 2:**
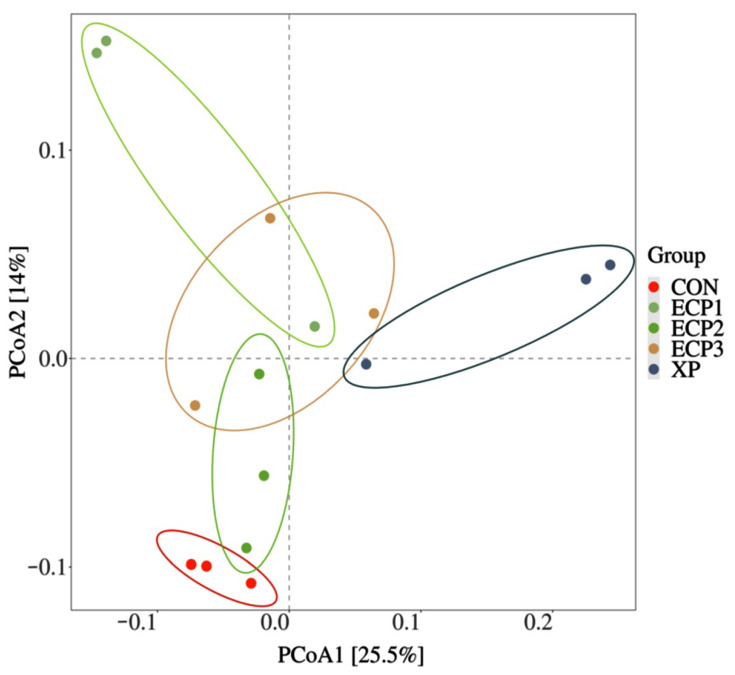
Bray–Curtis distance matrix PCoA of bacterial community in five treatments. CON treatment, without supplementation; XP treatment, XP yeast culture supplementation with 10 g/kg of substrate; ECP1, ECP supplementation with 6 g/kg of substrate, ECP2, ECP supplementation with 12 g/kg of substrate, ECP3, ECP supplementation with 18 g/kg of substrate.

To visualize the differences among the five treatments, we plotted a PCoA diagram with a Bray–Curtis distance matrix, as shown in Figure 2. The percentage of variation is represented by PCoA1 (25.5%) and PCoA2 (14.0%). The closer distances of samples with the same treatments in the plot indicated that the bacterial community compositions of the samples were similar. The separation among five treatments, indicating the presence of different bacterial structures.

### 3.6. Effect of ECP Supplementation on Bacterial Community

**Figure 3:**
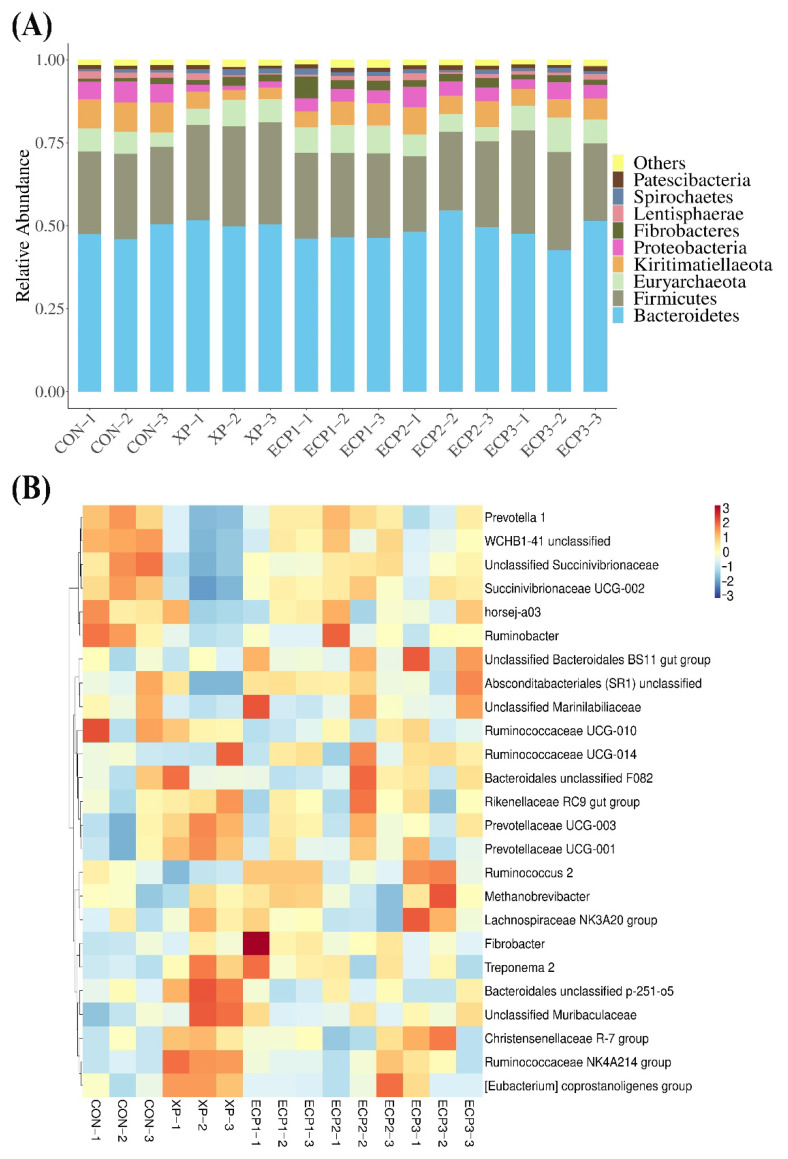
Rumen bacterial compositions at the phylum level (**A**) and heat map of species abundance at the genus level (**B**). CON, without supplementation; XP, XP yeast culture supplementation with 10 g/kg of substrate; ECP1, ECP supplementation with 6 g/kg of substrate, ECP2, ECP supplementation with 12 g/kg of substrate, ECP1, ECP3 supplementation with 18 g/kg of substrate.

A total of 30 phyla and 376 genera were isolated and taxonomically classified in the current study. The dominant phyla were Bacteroidetes (46.30–50.82%), followed by Firmicutes (24.09–28.06%), as shown in Table 6 and Figure 3A. The relative abundance of the phylum Firmicutes was higher in the XP treatment than in the CON treatment (*p* < 0.05) and was not affected by ECP supplementation compared with the CON treatment. The relative abundance of the phyla Kiritimatiellaeota and Proteobacteria were the highest value in the CON treatment. The relative abundance of the phylum Kiritimatiellaeota decreased linearly and quadratically with the ECP supplementation (*p* < 0.05). As shown in Table 6 and Figure 3B, the relative abundance of the phylum Fibrobacteres was higher in the ECP1 and ECP2 treatments than in the CON treatment (*p* < 0.05). The relative abundance of the genera *Prevotella 1*, *WCHB1-41 unclassified*, *Ruminococcus 2*, *Succinivibrionaceae UCG-002* and *Unclassified Succinivibrionaceae* were lower and the genera *Ruminococcaceae NK4A214 group* and *Lachnospiraceae NK3A20 group* were higher in the XP treatment than in the CON treatment (*p* < 0.05). The relative abundance of the genera *WCHB1-41 unclassified* and *Unclassified Succinivibrionaceaer* were lower and the genus *Ruminococcus 2* was higher in the ECP1 and ECP3 treatments than in the CON treatment (*p* < 0.05).

### 3.7. Prediction of Rumen Bacterial Functions

Based on the prediction of bacterial functions by the Tax4Fun program and the SILVA database, we selected gene functions from each group in annotation Level 2 for differential analysis. Supplementation with XP yeast culture or ECP significantly affected the prediction of rumen bacterial function. The main gene functions of rumen bacteria were associated with carbohydrate metabolism, amino acid metabolism and membrane transport, as shown in Table 7. The value of glycan metabolism and nucleotide metabolism were the lowest in the XP treatment. The value of nucleotide metabolism was lower in the ECP3 treatment than in the CON treatment (*p* < 0.05). The value of membrane transport and signal transduction were the highest in the XP treatment. The value of signal transduction was higher in the ECP3 treatment than in the CON treatment (*p* < 0.05). The value of cell motility was higher in the XP and ECP3 treatments than in the CON treatment (*p* < 0.05). Compared with the CON treatment, the value of cell growth and death was lower in the XP treatment (*p* < 0.05), and higher in the ECP1 treatment (*p* < 0.05). The values of replication and repair and of translation were lower in the XP and ECP3 treatments than in the CON treatment (*p* < 0.05). Compared with the CON treatment, the value of the digestive system was lower in the XP treatment (*p* < 0.05).

### 3.8. Correlation Analysis

**Figure 4:**
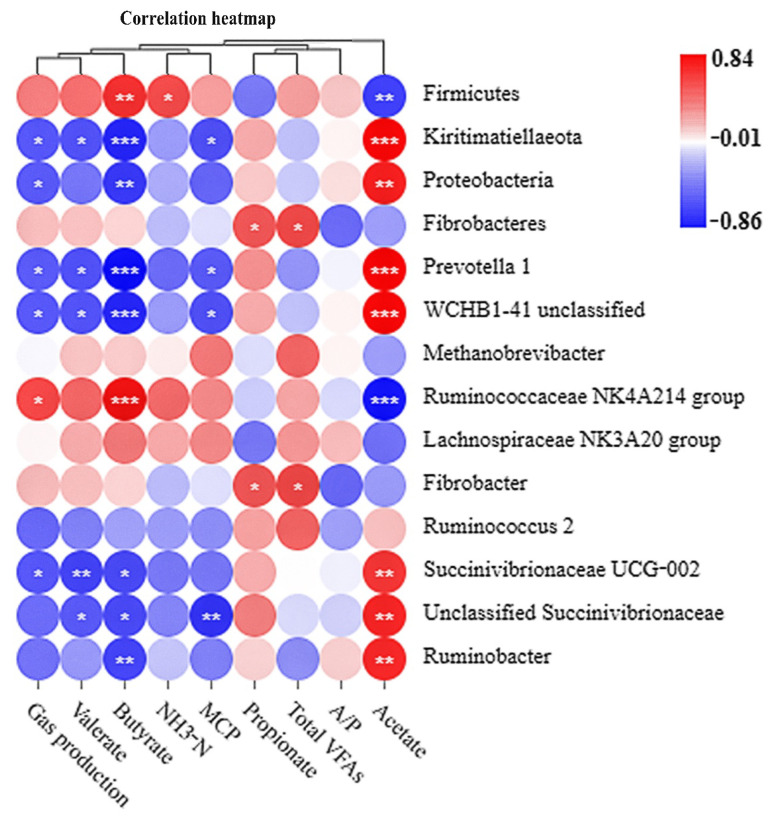
The correlation of gas production and rumen fermentation parameters with bacterial abundances in in vitro fermentation. The colors indicate positive (red, closer to 0.84) or negative (blue, closer to −0.86) correlations between gas production and rumen fermentation parameters with bacterial abundances. NH_3_-N, amine nitrogen; MCP, microbial protein; A/P, ration of acetate to propionate; VFAs, volatile fatty acids. *, *p* < 0.05; **, *p* < 0.01; ***, *p* < 0.001.

Correlations between gas production and rumen fermentation parameters with bacterial abundances in in vitro fermentation are as shown in Figure 4. Gas production was positively associated with the genus *Ruminococcaceae NK4A214 group* (r = 0.59 and *p* < 0.05), while negatively associated with the phyla Kiritimatiellaeota and Proteobacteria and the genera *Prevotella 1*, *WCHB1-41 unclassified*, and *Succinivibrionaceae UCG-002* (r < −0.56 and *p* < 0.05). The molar valerate proportion was correlated negatively with the phylum Kiritimatiellaeota and the genera *Prevotella 1*, *WCHB1-41 unclassified*, *Succinivibrionaceae UCG-002*, and *Unclassified Succinivibrionaceae* (r < −0.56 and *p* < 0.05). The molar butyrate proportion was positively associated with the phylum Firmicutes and the genus *Ruminococcaceae NK4A214 group* (r > 0.66 and *p* < 0.01) and negatively associated with the phyla Kiritimatiellaeota and Proteobacteria and the genera *Prevotella 1*, *WCHB1-41 unclassified*, *Succinivibrionaceae UCG-002*, *Unclassified Succinivibrionaceae*, and *Ruminobacter* (r < −0.62 and *p* < 0.05). The concentration of NH_3_-N was positively associated with the phylum Firmicutes (r = 0.57 and *p* < 0.05). The concentration of MCP was negatively associated with the phylum Kiritimatiellaeota and the genera *Prevotella 1*, *WCHB1-41 unclassified*, and *Unclassified Succinivibrionaceae* (r < −0.55 and *p* < 0.05). The molar propionate proportion and total VFAs concentration were positively associated with the phylum Fibrobacteres and the genus *Fibrobacter* (r > 0.50 and *p* < 0.01). The molar acetate proportion was positively associated with the phyla Kiritimatiellaeota and Proteobacteria and the genera *Prevotella 1*, *WCHB1-41 unclassified*, *Succinivibrionaceae UCG-002*, *Unclassified Succinivibrionaceae*, and *Ruminobacter* (r > 0.64 and *p* < 0.01) and negatively associated with the phylum Firmicutes and the genus *Ruminococcaceae NK4A214 group* (r < −0.55 and *p* < 0.01).

## 4. Discussion

Gas produced by in vitro fermentation is mainly derived from soluble carbohydrates [51]. The cumulative gas production gradually increased over time and was affected by different supplements at different times. More specifically, compared with the control group, supplementation of ECP with 12 g/kg of substrate increased the cumulative gas production at 2, 4, and 12 h; supplementation of XP yeast culture with 10 g/kg of substrate increased the cumulative gas production at 12, 24, and 36 h; supplementation of ECP with 18 g/kg of substrate increased the cumulative gas production at 48 h (Table 2). The data of cumulative gas production implied that supplemented ECP with 12 g/kg of substrate increased the gas production rate in the initial period, supplemented XP yeast culture with 10 g/kg of substrate increased the gas production rate in the medium-term, and supplemented ECP with 18 g/kg of substrate increased the late-stage gas production rate at the late stage during in vitro fermentation. Although there were no statistical differences in the kinetic parameters of gas production among the five treatments. The value of RahG (the average gas production rate at the time when half of the asymptotic gas production occurred) in the CON treatment was lower than in the other four treatments (2.9 vs. 3.2, 3.0, 3.2, 3.1). The yeast cultures used in this study contained yeast cell walls that were rich in glucans and mannans. These carbohydrates can be used as prebiotics to increase the activity of rumen microorganisms and to improve rumen fermentation [52]. Meanwhile, the glucans and mannans can be highly degraded in the rumen, producing more in vitro gas [53]. As a fermentation substrate, the rumen degradable amino acid content is another key factor affecting the growth rate of rumen microbes [54]. On the other hand, rumen available nitrogen in the form of peptides and amino acids can contribute to the digestion of structural carbohydrates and non-structural carbohydrates by rumen microbes [25]. Thus, it seems that ECP supplementation accelerated the fermentation and gas production processes, implicating that ECP or XP yeast culture supplementation enhanced the availability of soluble carbohydrates from substrates and that such beneficial effect was pronounced in the XP and ECP2 treatments.

Nitrogen metabolism in the rumen begins with the degradation of dietary protein or non-protein nitrogen by microorganisms into NH_3_-N, which is subsequently converted into MCP [55]. Microbial protein synthesized in the rumen is an important source of amino acids available for ruminants [56,57]. Compared with the control treatment, supplementation of yeast culture increased the NH_3_-N concentration at 24 h and the MCP concentration at 24 and 48 h in the incubation fluids; supplementation of ECP with 18 g/kg of substrate increased the NH_3_-N concentration at 12 and 36 h, increased the MCP concentration at 12, 36, and 48 h in the incubation fluids. Meanwhile, the NH_3_-N and MCP concentrations increased linearly and quadratically with the ECP supplementation at 36 h. The synthesis efficiency of MCP in the rumen is determined by the abundance of microbes that synthesize it and its ability to utilize NH_3_-N [51]. Both XP yeast culture and ECP could be used as a fermentation substrate so as to stimulate the growth of some ruminal microorganisms and improve the utilization of NH_3_-N.

Volatile fatty acids, the major end-products of rumen fermentation, are the main energy sources for ruminants. The composition and production of VFAs are important indicators of the rumen fermentation function [49,51]. For high lactation cows, acetate and butyrate produced in the rumen are substances for the synthesis of milk lipids, and 66.7% of the glucose used to produce lactose comes from gluconeogenesis from propionate [58,59]. The energy efficiency improved with the increased production of propionate and decreased production of acetate [60]. Because most carbohydrate fermentation by rumen bacteria results in a higher propionate concentration, an increase in propionate concentration often causes a drop in the ratio of acetate to butyrate in the rumen [61]. In this study, supplementation of XP yeast culture with 10 g/kg of substrate decreased the molar acetate proportion and increased the molar butyrate and valerate proportion at 48 h and decreased the ratio of acetate to proportion, suggesting that supplementation of XP yeast culture changed the fermentation pattern in vitro.

Previous studies have confirmed that the dominant phyla in rumen of ruminants are Firmicutes and Bacteroidetes [62], which is also supported by data from this study (Figure 3A and Table 6). This illustrated that the bacterial composition and proportion of the in vitro fermentation system in this study was the same as that of the cow rumen. The Bacteroidetes in rumen are active in the degradation of non-cellulosic plant constituent and the production of acetate and propionate [63]. The main members of the phylum Firmicutes are diverse fibrolytic and cellulolytic bacterial genera that are involved in the degradation of cellulose, hemicellulose, oligosaccharides, and starch, improving the digestion and utilization of fibers in ruminants [64]. The phyla Kiritimatiellaeota and Proteobacteria have been suggested to be involved in the degradation of fiber and organic matter, respectively [64]. Supplementation of XP yeast culture with 10 g/kg of substrate increased the relative abundance of the phylum Firmicutes while decreased the relative abundance of the phyla Kiritimatiellaeota and Proteobacteria, compared with the control group. Supplementation of ECP with 6 and 18 g/kg of substrate decreased the relative abundance of the phyla Kiritimatiellaeota and Proteobacteria while supplementation of ECP with 6 and 12 g/kg of substrate increased the relative abundance of the phylum Fibrobacteres, compared with the control group. The results of correlation analysis showed that the phylum Firmicutes was positively associated with the molar butyrate proportion while negatively associated with the molar butyrate proportion. In contrast, the phyla Kiritimatiellaeota and Proteobacteria were negatively associated with the molar butyrate proportion while positively associated with the molar butyrate proportion. These results indicated that supplementation with XP yeast culture or ECP had different effects on microbial diversity during in vitro fermentation.

Members of the genus *Prevotella* are considered to be the most abundant group of ruminal bacteria [65,66], and several enzymes involved in fiber digestion have been identified in members of this genus [67,68]. In this study, the genus *Prevotella 1* was positively associated with the molar acetate proportion while negatively associated with gas production, MCP concentration, and the molar proportions of butyrate and valerate. The low abundance of *Prevotella 1* could be the reason for the low molar acetate proportion in the XP treatment. The lowest function percentage of energy metabolism and concentration of total VFAs in the XP treatment suggested that supplementation of XP yeast culture with 10 g/kg of substrate may not be beneficial to energy metabolism activities of rumen bacteria. The members of the family Ruminococcaceae are another major component of Firmicutes in the degradation of fiber [69]. The genus *Ruminococcaceae NK4A214 group* belongs to the family Ruminococcaceae, and although it was first identified and cultured in 2011 [70], its precise function in the rumen is still not clear [71]. In this study, the genus *Ruminococcaceae NK4A214 group* was positively associated with gas production and the molar butyrate proportion while negatively associated with the molar acetate proportion. Previous study has shown that high dietary soluble carbohydrates increased the content of butyrate in the rumen of dairy cows [72]. Thus, we speculate that the function of the genus *Ruminococcaceae NK4A214 group* may be related to degradation of soluble carbohydrates. Although accounting for a relatively small proportion, the high fibrolytic activity makes genera *Fibrobacter* and *Ruminococcus* play a nonnegligible role in fiber degradation [73]. Supplementation of ECP with 6 g/kg of substrate increased the relative abundance of the genera *Fibrobacter* and *Ruminococcus*, which could result in the increase of the concentration of total VFAs at 24 h (63.8 vs. 71.6 mmol/L). In fact, microbes that account for a larger proportion of abundance contribute significantly to the function of the rumen microbial ecosystem, and the small group of bacteria in the rumen community may possess important but as yet unrecognized ecological functions [69]. One of the limitations of this study is the low number of repetitions, which should be noted in future experiments. Meanwhile, the bacterial diversity at 24 h may be not enough to reveal the relationship between bacteria and rumen fermentation parameters.

## 5. Conclusions

This study provided information about in vitro dynamic changes of ruminal fermentation characteristics, the kinetic of gas production, and microbial diversity with different levels of ECP supplementation. Overall, the molar propionate proportion at 12 and 24 h was the highest in the ECP1 treatment. The cumulative gas production at 2 and 4 h was the highest in the ECP2 treatment. The cumulative gas production at 12 and 48 h, the concentration of NH_3_-N at 12 and 36 h, the concentration of MCP at 12, 36, and 48 h, and the NGR value were the highest in the ECP3 treatment. In addition, supplementation of ECP with 6 or 18 g/kg of substrate increased the relative abundance of *Ruminococcus 2* and decreased the relative abundance of *WCHB1-41 unclassified* and *Unclassified Succinivibrionaceae.* Supplementation of ECP had no negative impact on gas production, in vitro fermentation characteristics, and microbial diversity, and the level of ECP in vitro experiment was recommended to be 18 g/kg; however, in vivo feeding trials are needed in order to assess the best ECP supplementation dose.

## Figures and Tables

**Table 1:** Ingredients and chemical composition of the fermentation substrate in vitro (DM basis).

Ingredients	%	Chemical Composition	%
Corn	66.34	Crude protein	11.62
Rapeseed meal	8.05	Neutral detergent fiber	18.81
Distillers’ grains	9.16	Acid detergent fiber	9.72
Wheat bran	7.96	Calcium	0.49
Sodium bicarbonate	1.70	Phosphorus	0.37
Sodium chloride	1.13		
Premix ^1^	5.66		
Total	100.00		

^1^ Premix provide per kg of the fermentation substrate with: Cu 10 mg, Fe 50 mg, Mn 20 mg, Zn 30 mg, Se 0.1 mg, I 0.5 mg, Co 0.1 mg, VA 2200 IU, VD 275 IU, VE 50 IU.

**Table 2:** In vitro kinetic parameters under different levels of ECP supplementation ^1^.

Items	Treatments ^2^					SEM	*p*-Value ^3^		
	CON	XP	ECP1	ECP2	ECP3		T	L	Q
Cumulative gas production (mL/0.2 g DM)									
2 h	7.3 ^a^	7.6 ^ab^	7.6 ^ab^	9.3 ^c^	8.3 ^b^	0.21	0.001	0.034	0.033
4 h	17.1 ^ab^	18.7 ^bc^	17.0 ^ab^	19.6 ^c^	15.5 ^a^	0.46	0.009	0.633	0.136
8 h	34.4	33.7	34.1	34.8	33.3	0.41	0.843	0.552	0.712
12 h	39.6 ^a^	42.9 ^b^	40.5 ^a^	44.5 ^b^	46.9 ^b^	0.70	<0.001	<0.001	<0.001
24 h	52.8 ^a^	60.5 ^b^	55.1 ^ab^	55.1 ^ab^	56.1 ^ab^	0.90	0.074	0.006	0.009
36 h	58.2	61.6	59.1	60.3	59.00	0.47	0.130	0.388	0.349
48 h	59.7 ^a^	62.2 ^ab^	60.4 ^a^	61.2 ^ab^	67.9 ^b^	1.15	0.045	0.040	0.064
Kinetic parameters									
A (mL/0.2 g DM)	64.2	68.1	65.1	66.1	71.6	1.50	0.610	0.179	0.350
B	1.4	1.5	1.4	1.4	1.5	0.04	0.907	0.574	0.834
C (h)	7.9	7.9	7.9	7.3	9.1	0.36	0.696	0.510	0.528
TRmaxG (mL/h)	2.3	2.7	2.5	2.1	2.7	0.13	0.602	0.655	0.756
RmaxG (h)	3.4	3.5	3.4	3.9	3.4	0.08	0.174	0.666	0.458
RahG (mL/h)	2.9	3.2	3.0	3.2	3.1	0.12	0.883	0.471	0.724

^1^ Means in the same row followed by different letters differ (*p* < 0.05); ECP, enzymatic hydrolysate of cottonseed protein; A, the asymptotic gas production (mL/0.2 g DM); B, the sharpness parameter determining the shape of the curve; C, the time (h); TRmaxG, the time corresponding to the maximum rate of gas production); RmaxG, the maximum gas production rate; RahG, the average gas production rate at the time when half of “A” occurred. ^2^ CON, XP, ECP1, ECP2 and ECP3 (without supplementation, XP yeast culture supplementation with10 g/kg of substrate; ECP supplementation with 6, 12, 18 g/kg of substrate). ^3^ T = treatment, comparison among five treatments; L = linear, linear effect of different ECP supplementation levels; Q = quadratic, quadratic effect of different ECP supplementation levels.

**Table 3:** Concentrations of NH_3_-N and MCP under different levels of ECP supplementation ^1^.

Items	Treatments ^2^					SEM	*p*-Value ^3^		
	CON	XP	ECP1	ECP2	ECP3		T	L	Q
NH_3_-N (mg/L)									
8 h	72.5	82.8	50.7	60.7	55.4	4.22	0.070	0.307	0.396
12 h	91.4 ^a^	110.4 ^ab^	79.4 ^a^	88.1 ^a^	149.2 ^b^	8.32	0.025	0.024	0.002
24 h	216.7 ^a^	310.2 ^b^	194.1 ^a^	220.8 ^a^	239.8 ^a^	13.38	0.029	0.106	0.060
36 h	288.0 ^a^	319.6 ^bc^	306.3 ^ab^	324.1 ^bc^	331.0 ^c^	4.80	0.008	<0.001	0.002
48 h	341.1	375.2	351.9	376.8	395.8	10.05	0.495	0.087	0.246
MCP (mg/L)									
8 h	111.9	111.5	95.7	105.7	107.9	2.88	0.420	0.951	0.454
12 h	100.4 ^a^	121.2 ^ab^	130.2 ^b^	119.0 ^ab^	131.7 ^b^	3.86	0.041	0.047	0.091
24 h	95.5 ^a^	136.8 ^b^	100.5 ^a^	97.4 ^a^	108.0 ^a^	4.43	0.001	0.148	0.321
36 h	72.4 ^a^	89.7 ^ab^	88.4 ^ab^	89.9 ^ab^	112.7 ^b^	4.41	0.037	0.006	0.024
48 h	81.5 ^a^	104.0 ^bc^	94.8 ^ab^	85.1 ^a^	115.4 ^c^	3.76	0.003	0.006	0.010

^1^ Means in the same row followed by different letters differ (*p* < 0.05); ECP, enzymatic hydrolysate of cottonseed protein; NH_3_-N, ammonia nitrogen; MCP, microbial protein. ^2^ CON, XP, ECP1, ECP2, and ECP3 (without supplementation, XP yeast culture supplementation with10 g/kg of substrate; ECP supplementation with 6, 12, 18 g/kg of substrate). ^3^ T = treatment, comparison among five treatments; L = linear, linear effect of different ECP supplementation levels; Q = quadratic, quadratic effect of different ECP supplementation levels.

**Table 4:** Composition and production of VFAs under different levels of ECP supplementation ^1^.

Items	Treatments ^2^					SEM	*p*-Value ^3^		
	CON	XP	ECP1	ECP2	ECP3		T	L	Q
Acetate (%, molar)									
12 h	66.59 ^c^	63.86 ^a^	65.47 ^b^	65.85 ^b^	65.45 ^b^	0.25	<0.001	0.034	0.441
24 h	66.44	64.28	65.06	66.54	64.92	0.37	0.223	0.051	0.748
48 h	66.04 ^b^	63.93 ^a^	65.94 ^b^	65.45 ^ab^	64.60 ^ab^	0.26	0.013	0.001	0.003
Propionate (%, molar)									
12 h	15.86 ^ab^	16.49 ^bc^	16.85 ^c^	16.06 ^ab^	15.55 ^a^	0.14	0.007	0.280	0.030
24 h	15.68 ^ab^	14.68 ^a^	16.36 ^b^	15.93 ^b^	15.52 ^ab^	0.19	0.045	0.403	0.025
48 h	16.15	16.03	16.00	15.94	15.69	0.13	0.899	0.257	0.535
Butyrate (%, molar)									
12 h	16.60 ^a^	18.62 ^c^	16.75 ^a^	17.11 ^a^	17.93 ^b^	0.21	<0.001	<0.001	<0.001
24 h	16.78 ^a^	19.62 ^c^	17.41 ^ab^	16.09 ^a^	18.27 ^ab^	0.43	0.048	0.426	0.502
48 h	16.46 ^a^	18.42 ^b^	16.66 ^a^	17.11 ^a^	18.15 ^b^	0.23	<0.001	<0.001	<0.001
Valerate (%, molar)									
12 h	0.95	1.03	0.93	0.98	1.07	0.02	0.077	0.026	0.044
24 h	1.10	1.42	1.17	1.44	1.29	0.05	0.164	0.130	0.221
48 h	1.35 ^a^	1.62 ^d^	1.40 ^ab^	1.50 ^bc^	1.56 ^cd^	0.03	0.001	0.001	0.004
Total VFA (mmol/L)									
12 h	57.6 ^b^	51.9 ^a^	60.1 ^b^	62.1 ^b^	57.4 ^b^	1.06	0.006	0.822	0.094
24 h	63.8	65.1	71.6	65.2	70.1	2.19	0.608	0.864	0.986
48 h	69.5	72.3	80.4	82.9	78.2	2.66	0.115	0.220	0.014
A/P									
12 h	4.2 ^b^	3.8 ^a^	3.9 ^a^	4.1 ^b^	4.2 ^b^	0.18	0.005	0.594	0.065
24 h	4.2	4.4	4.0	4.6	4.3	0.23	0.299	0.922	0.464
48 h	4.2	4.5	4.1	4.1	4.4	0.16	0.901	0.861	0.985
NGR									
12 h	6.0 ^bc^	5.8 ^ab^	5.6 ^a^	5.9 ^bc^	6.2 ^c^	0.06	0.012	0.220	0.024
24 h	6.0 ^a^	6.6 ^b^	5.8 ^a^	6.2 ^ab^	6.2 ^ab^	0.09	0.024	0.603	0.001
48 h	5.9	6.4	5.8	5.8	6.4	0.05	0.831	0.227	0.455

^1^ Means in the same row followed by different letters differ (*p* < 0.05); ECP, enzymatic hydrolysate of cottonseed protein; VFA, volatile fatty acid; A/P, the ratio of acetate to propionate; NGR, the ratio of non-glucogenic to glucogenic acids. ^2^ CON, XP, ECP1, ECP2, and ECP3 (without supplementation, XP yeast culture supplementation with 10 g/kg of substrate; ECP supplementation with 6, 12, 18 g/kg of substrate). ^3^ T = treatment, comparison among five treatments; L = linear, linear effect of different ECP supplementation levels; Q = quadratic, quadratic effect of different ECP supplementation levels.

**Table 5:** The sequencing data information and alpha diversity analysis ^1^.

Items	Treatments ^2^					SEM	*p*-Value ^3^		
	CON	XP	ECP1	ECP2	ECP3		T	L	Q
OTUs	1765 ^a^	2184 ^ab^	2342 ^b^	1761 ^a^	1806 ^ab^	90.05	0.089	0.628	0.390
Chao1 index	2805.9	2754.1	3155.9	2093.0	2142.9	161.5	0.609	0.611	0.483
Simpson index	0.994 ^b^	0.991 ^a^	0.994 ^b^	0.993 ^ab^	0.994 ^b^	0.001	0.064	0.924	0.915
Shannon index	6.2251	6.3206	6.4157	6.2199	6.2787	0.032	0.182	0.637	0.637

^1^ Means in the same row followed by different letters differ (*p* < 0.05); OUTs, operational taxonomic units. ^2^ CON, XP, ECP1, ECP2, and ECP3 (without supplementation, XP yeast culture supplementation with 10 g/kg of substrate; ECP supplementation with 6, 12, 18 g/kg of substrate, respectively). ^3^ T = treatment, comparison among five treatments; L = linear, linear effect of different ECP supplementation levels; Q = quadratic, quadratic effect of different ECP supplementation levels.

**Table 6:** Relative abundance of bacteria under different levels of ECP supplementation ^1^.

Items	Treatments ^2^					SEM	*p*-Value ^3^		
	CON	XP	ECP1	ECP2	ECP3		T	L	Q
Phylum (%)									
Bacteroidetes	47.98	50.65	46.30	50.82	47.21	2.98	0.229	0.798	0.504
Firmicutes	24.6 ^a^	29.8 ^b^	25.6 ^ab^	24.1 ^ab^	28.1 ^ab^	2.89	0.037	0.190	0.103
Euryarchaeota	5.97	6.61	8.17	5.39	8.31	1.67	0.053	0.372	0.570
Kiritimatiellaeota	8.90 ^c^	3.86 ^a^	6.23 ^b^	7.17 ^bc^	5.68 ^ab^	1.92	0.001	0.018	0.027
Proteobacteria	5.72 ^c^	1.78 ^a^	3.89 ^b^	4.85 ^bc^	4.00 ^b^	1.52	0.001	0.132	0.328
Fibrobacteres	1.31 ^a^	1.99 ^ab^	3.94 ^c^	2.37 ^b^	1.68 ^a^	1.30	0.088	0.907	0.661
Genus (%)									
*Prevotella 1*	10.36 ^b^	6.62 ^a^	8.78 ^ab^	9.83 ^ab^	7.89 ^ab^	1.57	0.016	0.038	0.124
*WCHB1-41 unclassified*	8.90 ^c^	3.86 ^a^	6.23 ^b^	7.17 ^bc^	5.68 ^ab^	1.92	0.001	0.018	0.050
*Methanobrevibacter*	5.16	5.82	7.04	4.62	7.27	1.47	0.084	0.351	0.608
*Ruminococcaceae NK4 A214 group*	2.59 ^a^	5.23 ^b^	3.01 ^a^	3.38 ^a^	3.37 ^a^	1.09	0.006	0.147	0.320
*Lachnospiraceae NK3A20 group*	2.96 ^ab^	3.50 ^b^	3.38 ^b^	2.51 ^a^	3.85 ^b^	0.62	0.040	0.310	0.298
*Fibrobacter*	1.30	1.97	3.93	2.35	1.66	1.30	0.088	0.902	0.128
*Ruminococcus 2*	1.52 ^b^	1.09 ^a^	1.91 ^c^	1.34 ^ab^	1.92 ^c^	0.39	0.026	0.494	0.721
*Succinivibrionaceae UCG-002*	1.69 ^c^	0.37 ^a^	1.23 ^bc^	1.39 ^bc^	1.20 ^b^	0.50	0.001	0.077	0.153
*Unclassified Succinivibrionaceae*	1.95 ^c^	0.28 ^a^	1.08 ^b^	1.51 ^bc^	1.09 ^b^	0.61	<0.001	0.049	0.095
*Ruminobacter*	1.43	0.25	0.50	1.04	0.57	0.57	0.058	0.179	0.333

^1^ Means in the same row followed by different letters differ (*p* < 0.05); ECP, enzymatic hydrolysate of cottonseed protein; VFA, volatile fatty acid; A/P, the ratio of acetate to propionate; NGR, the ratio of non-glucogenic to glucogenic acids. ^2^ CON, XP, ECP1, ECP2, and ECP3 (without supplementation, XP yeast culture supplementation with 10 g/kg of substrate; ECP supplementation with 6, 12, 18 g/kg of substrate). ^3^ T = treatment, comparison among five treatments; L = linear, linear effect of different ECP supplementation levels; Q = quadratic, quadratic effect of different ECP supplementation levels.

**Table 7:** The prediction of rumen bacterial function (%) ^1^.

Items	Treatments ^2^					SEM	*p*-Value ^3^		
	CON	XP	ECP1	ECP2	ECP3		T	L	Q
Metabolism									
Lipid metabolism	2.78	2.81	2.80	2.80	2.77	0.006	0.262	0.741	0.119
Energy metabolism	6.91	6.88	6.94	6.91	6.93	0.008	0.108	0.202	0.453
Amino acid metabolism	11.48	11.50	11.56	11.45	11.58	0.024	0.389	0.349	0.631
Glycan metabolism	4.05 ^b^	3.77 ^a^	3.95 ^ab^	4.07 ^b^	3.86 ^ab^	0.039	0.035	0.765	0.940
Carbohydrate metabolism	15.41	15.43	15.42	15.52	15.35	0.025	0.335	0.883	0.467
Nucleotide metabolism	7.13 ^c^	6.87 ^a^	7.10 ^bc^	7.08 ^bc^	7.03 ^b^	0.026	<0.001	0.917	0.787
Environmental information processing									
Membrane transport	8.28 ^ab^	8.94 ^c^	8.39 ^ab^	8.18 ^a^	8.67 ^bc^	0.089	0.011	0.983	0.925
Signal transduction	6.41 ^a^	6.92 ^c^	6.42 ^a^	6.43 ^a^	6.65 ^b^	0.058	0.001	0.966	0.988
Cellular processes									
Transport and catabolism	0.37	0.36	0.36	0.38	0.36	0.004	0.518	0.876	0.983
Cell motility	1.71 ^a^	2.04 ^c^	1.73 ^ab^	1.69 ^a^	1.89 ^bc^	0.041	0.003	0.992	0.987
Cell growth and death	1.82 ^b^	1.80 ^a^	1.84 ^c^	1.82 ^b^	1.82 ^b^	0.004	0.004	0.458	0.731
Genetic information processing									
Replication and repair	6.03 ^c^	5.81 ^a^	6.00 ^bc^	6.03 ^c^	5.88 ^ab^	0.028	0.005	0.718	0.939
Translation	6.34 ^c^	6.11 ^a^	6.27 ^bc^	6.33 ^c^	6.18 ^ab^	0.027	0.005	0.684	0.888
Organismal systems									
Endocrine system	0.39	0.38	0.39	0.40	0.38	0.003	0.120	0.625	0.886
Environmental adaptation	0.25	0.25	0.25	0.25	0.25	0.001	0.325	0.451	0.436
Digestive system	0.44 ^b^	0.37 ^a^	0.41 ^ab^	0.44 ^b^	0.39 ^ab^	0.010	0.032	0.703	0.754

^1^ Means in the same row followed by different letters differ (*p* < 0.05). ^2^ CON, XP, ECP1, ECP2, and ECP3 (without supplementation, XP yeast culture supplementation with 10 g/kg of substrate; ECP supplementation with 6, 12, 18 g/kg of substrate). ^3^ T = treatment, comparison among five treatments; L = linear, linear effect of different ECP supplementation levels; Q = quadratic, quadratic effect of different ECP supplementation levels.

## Data Availability

All data generated, analyzed, or used in this study are available from the relevant authors on reasonable request. The datasets generated for this study can be found online at: https://www.ncbi.nlm.nih.gov/bioproject/PRJNA850887, accessed on 22 June 2022.

## Supplementary Materials

The following supporting information can be downloaded at: https://www.mdpi.com/article/10.3390/ani12162113/s1, Figure S1: Multi samples rarefaction curves; Table S1: The sequencing data information of bacteria.
