# Supplementary material for: Rumen Fermentation and Microbiome Responses to Enzymatic Hydrolysate of Cottonseed Protein Supplementation in Continuous In Vitro Culture
Source: Animals (Basel). 2022 Aug 18;12(16):2113. doi: 10.3390/ani12162113 (PMC9405472; doi:10.3390/ani12162113)
Supplement: Supplementary file 1 [file animals-12-02113-s001.zip › animals-1832275-supplementary.pdf]

Table S1 The sequencing data information of bacteria

Samples No.	Raw reads	Effective reads	Effective rate (%)	OTU counts	Q30(%)
CON_1	34376	29985	87.23	1785	94.29
CON_2	39009	33847	86.77	1777	93.79
CON_3	38234	34750	90.89	1733	93.22
XP_1	40647	37943	93.35	1786	94.76
XP_2	31771	29280	92.16	2298	94.50
XP_3	34642	31148	89.91	2468	94.57
ECP1_1	34120	29498	86.45	1736	93.51
ECP1_2	41640	38170	91.67	2618	94.39
ECP1_3	33315	30407	91.27	2673	94.53
ECP2_1	36800	33959	92.28	1800	93.85
ECP2_2	34958	31610	90.42	1711	93.27
ECP2_3	38814	35912	92.52	1772	93.18
ECP3_1	40125	37203	92.72	1808	95.00
ECP3_2	41036	37797	92.11	1776	94.46
ECP3_3	34072	31408	92.18	1834	93.72

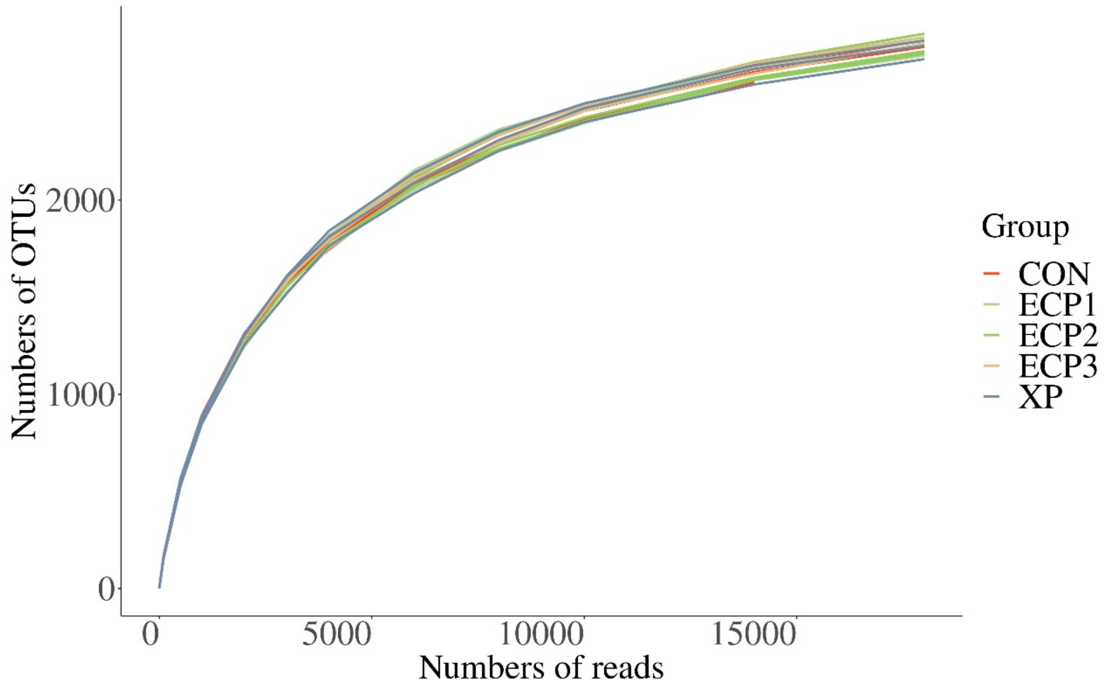

Figure S1. Multy samples Rarefaction Curves
